# Clinical Characteristics, Risk Factors for Severity and Pharmacotherapy in Hospitalized COVID-19 Patients in the United Arab Emirates

**DOI:** 10.3390/jcm11092439

**Published:** 2022-04-26

**Authors:** Amna Mohamed Juma Almarashda, Syed Arman Rabbani, Martin Thomas Kurian, Ajith Cherian

**Affiliations:** 1 Kalba Hospital, Sharjah P.O. Box 11195, United Arab Emirates; amna.taffak@ehs.gov.ae; 2 Department of Clinical Pharmacy and Pharmacology, RAK College of Pharmacy, RAK Medical and Health Sciences University, Ras Al Khaimah P.O. Box 11172, United Arab Emirates; 3 Department of Nephrology, Ibrahim Bin Hamad Obaidallah Hospital, Ras Al Khaimah P.O. Box 4727, United Arab Emirates; martin.kurian@ehs.gov.ae; 4 Department of Internal Medicine, Ibrahim Bin Hamad Obaidallah Hospital, Ras Al Khaimah P.O. Box 4727, United Arab Emirates; ajith.cherian@ehs.gov.ae

**Keywords:** COVID-19, clinical characteristics, biomarkers, risk factors, severity, pharmacotherapy, United Arab Emirates

## Abstract

Data on the clinical characteristics, severity and management of COVID-19 from the Middle East region, especially the United Arab Emirates (UAE), is very limited. We studied the clinical characteristics, laboratory biomarkers, risk factors for severity and pharmacotherapy of hospitalized COVID-19 patients in this single-center, analytical cross-sectional study conducted in a secondary care hospital of the UAE. A total of 585 patients were included in the study (median age, 49 years (IQR, 39–59); 66% male). Age > 45 years (OR = 2.07, 95% CI: 1.04–4.14, *p* = 0.040), male gender (OR = 3.15, 95% CI: 1.52–6.51, *p* = 0.002), presentation symptoms such as fever (OR = 3.68, 95% CI:1.34–10.11, *p* = 0.011) and shortness of breath/dyspnea (OR = 5.36, 95% CI: 2.69–10.67, *p* < 0.001), Hb < 13 g/dL (OR = 3.17, 95% CI: 1.51–6.65, *p* = 0.002), neutrophils > 7 × 10^3^/mcL (OR = 4.89, 95% CI: 1.66–14.37, *p*=0.004), lymphocytes < 1 × 10^3^/mcL (OR = 7.78, 95% CI: 1.01–60.19, *p* = 0.049), sodium < 135 mmol/L (OR = 5.42, 95% CI: 1.05–27.95, *p* = 0.044), potassium < 3.6 mmol/L (OR = 3.36, 95% CI: 1.03–11.01, *p* = 0.045), urea > 6.5 mmol/L (OR = 3.37, 95% CI: 1.69–6.73, *p* = 0.001) and LDH > 227 IU/L (OR = 6.26, 95% CI: 1.61–24.32, *p* = 0.008) were independent predictors of the severity of COVID-19. Antivirals (524, 89.6%) and corticosteroids (358, 61.2%) were prescribed for the management of COVID-19. In conclusion, older age, male gender, presentation symptoms such as fever and dyspnea, low hemoglobin, neutrophilia, lymphopenia, hyponatremia, hypokalemia, elevated levels of urea and lactate dehydrogenase were found to be independent risk factors for severe COVID-19. The pharmacotherapy of COVID-19 patients in our study was diverse, and the medications were prescribed based on the clinical condition of the patients.

## 1. Introduction

Coronavirus disease 2019 (COVID-19), caused by severe acute respiratory syndrome coronavirus 2 (SARS-CoV-2), has exponentially spread to almost all the countries in the world [1] and has posed unprecedented challenges to the healthcare system. Globally, as of 14 March 2022, more than 458 million cases of COVID-19 have been reported, including more than 6 million deaths [2]. In the United Arab Emirates (UAE), around 885 thousand COVID-19 cases and 2302 deaths have been reported as of 14 March 2022 [3].

People of all ages are at risk of contracting the disease. However, older-aged people and those with chronic medical conditions have a higher probability of developing severe disease [4]. The clinical presentation of the disease is heterogeneous and can range from asymptomatic to severe disease with pneumonia, acute respiratory distress syndrome and even death [4,5,6]. Studies have shown that COVID-19 can also lead to neurologic, cardiovascular, hematologic, gastrointestinal, dermatologic, musculoskeletal, renal and hepatic complications. Thromboembolic complications have also been reported in patients with COVID-19 [7,8].

A number of vaccines such as BNT162b2 (Pfizer-BioNTech), mRNA-1273 (Moderna), Ad26.COV2.S (Johnson & Johnson/Janssen), AZD1222 (Oxford/AstraZeneca), etc. have been given approvals and emergency use authorizations in different countries around the world [9]. The Ministry of Health and Prevention, UAE has also approved four vaccines for use in eligible individuals against the COVID-19 infection, vaccines by Sinopharm, Pfizer-BioNTech, Sputnik V, Oxford-AstraZeneca and Moderna [10].

The spectrum of pharmacotherapy for COVID-19 management is rapidly changing and evolving. A range of drugs used for other indications and many investigational agents is still being evaluated in clinical trials for their roles in the management of COVID-19 [11,12,13]. Currently, the mainstay of management includes prevention and control of infection and supportive care, with supplemental oxygen and mechanical ventilation [11,13].

Country-specific guidelines, varying from country to country, have been published all over the world with recommendations for COVID-19 management [13,14,15]. In the UAE also, the National Clinical Committee for COVID-19 Management, Ministry of Health and Prevention has published guidance on the clinical management of COVID-19 [14].

Presently, the number of COVID-19 cases and deaths has dropped significantly, globally [16] as well as in the UAE [17]. In the UAE, this drop is attributed to high COVID-19 vaccination rates. In addition to this, compliance with precautionary measures and strengthened medical facilities have resulted in enhanced recovery and reduced mortality rates. However, given the emerging new variants of SARS-CoV-2 and the rapidity with which the COVID-19 landscape is evolving, this pandemic is far from over. It is very important to make well-timed diagnoses and initiate the effective management of patients based on the severity of the disease. Therefore, the present study investigated the clinical characteristics, risk factors for severity and pharmacotherapy of COVID-19 patients admitted in a secondary care hospital in the UAE.

## 2. Materials and Methods

### 2.1. Study Population and Setting

This single-center, analytical cross-sectional study was conducted at Ibrahim Bin Hamad Obaidallah Hospital, which is a COVID-19 dedicated hospital in the emirate of Ras Al Khaimah, UAE. It is a secondary care multispecialty center with internal medicine, nephrology, neurology, cardiology, gastroenterology, geriatrics, psychiatry and emergency medicine departments. Patients with positive real-time, reverse transcriptase–polymerase chain reaction (RT-PCR) tests for SARS-CoV2 and admitted to the study site between 1 April 2020 and 31 April 2021 were included in the study. The minimum sample size, considering the proportion of patients with severe illness 14% [18], with a 95% confidence level and a 3% margin of error, was 514.

### 2.2. Severity of COVID-19

The study population was stratified into four groups as per the severity of illness at the time of admission in accordance with the National Guidelines for the Clinical Management and Treatment of COVID-19, Ministry of Health and Prevention, UAE [14]: asymptomatic, mild, moderate, severe and critical. The first three groups, asymptomatic, mild and moderate, were further grouped together as a non-severe group, and the latter two, severe and critical, as the severe group.

### 2.3. Data Collection

Patient data were collected from the electronic medical records and included epidemiological information, clinical characteristics, laboratory investigations and treatment regiments. All the collected data were reviewed by the study investigators to ensure completeness. All measures were taken to preserve the integrity and confidentiality of the patient data.

### 2.4. Statistical Analysis

Study data was analyzed using Statistical Package for the Social Sciences (SPSS) version 27.0. Skewness and kurtosis were checked before the analysis. The normality of the data distribution was tested using Shapiro–Wilk test. Frequency and percentages with 95% confidence intervals (CIs) were used to describe categorical variables. The median and interquartile range (IQR) with 95% CIs were used for continuous variables. A chi square test or Fisher’s exact test was used for comparing categorical variables, whereas a two-sample median test was used to compare continuous variables. Patients were stratified as per the severity of COVID-19 into two groups: severe and non-severe. The multiple imputation technique was used for handling missing data. Logistic regression models, univariable and multivariable, were used to identify the risk factors associated with COVID-19 severity. The odds ratios (OR) along with the 95% CIs were reported. All statistical tests were 2-tailed, and *p* < 0.05 was considered statistically significant.

### 2.5. Ethics

The study was approved by the Ras Al Khaimah Medical and Health Sciences University Research and Ethics Committee (Number: RAKMHSU-REC-012-2020/21-F-P) and the Ministry of Health and Prevention Research Ethics Committee/RAK Subcommittee (MOHAP/REC/2020/56-2020-F-P). Additionally, the study received approvals from the Medical Director of Ibrahim Bin Hamad Obaidallah Hospital and Undersecretary of Hospital Sector, Ministry of Health and Prevention, UAE.

## 3. Results

### 3.1. Socio-Demographic and Clinical Characteristics

A total of 585 patients with COVID-19 who were hospitalized at the study site during the study period were included in the study. Out of these patients, 386 (66%) were males and 394 (67%) were non-Arabs. The median age of the study population was 49 years (IQR, 39–59) with a majority of patients in the age group of 41–60 years (282, 48.2%). The median body mass index (BMI) of the study patients was 28.0 kg/m^2^ (IQR, 25–33). One hundred and forty-four (25%) patients had a history of contact with a known COVID-19 case whereas 441 patients had no clear history of direct contact. Only a fraction of patients in our study were alcoholics (26, 4.4%) and smokers (38, 6.5%). Majority of the patients had one to two comorbidities (267, 45.6%), with diabetes being the most common comorbid condition (234, 40.0%), followed by hypertension (214, 36.6%) and cardiovascular disease (119, 20.3%). The median length of hospital stay in our study was 9 days (IQR, 6–14) with a maximum of 60 days and a minimum of 1 day.

Regarding the severity of the disease, the majority of the COVID-19 patients in our study had moderate disease (229, 39.1%), followed by mild (165, 28.2%), severe (116, 19.8%) and critical (38, 6.5%) disease. One hundred and seventy-three patients (30%) were admitted to intensive care unit for the management of their condition, and only 116 (19.8%) patients were intubated and received mechanical ventilation. The most common symptoms on presentation to the hospital were fever (465, 79.5%) and dry cough (417, 71.3%), followed by shortness of breath (316, 54 %), myalgia (126, 21.5 %) and nausea or vomiting (80, 13.7%).

A significantly higher proportion of patients over the age of 45 years was in the severe group as compared to the non-severe group (67.5% vs. 57.3%, *p* = 0.026). In addition to this, the severe group had a significantly higher proportion of males than the non-severe group (76.6% vs. 62.2%, *p* = 0.001). Furthermore, patients with a higher number of underlying diseases were more likely to develop severe symptoms. The severe group had a significantly higher proportion of patients with more than two comorbidities than the non-severe group (26.6 % vs. 17.9%, *p* = 0.035). The proportions of patients having diabetes as a comorbid condition was significantly higher in the severe group than the non-severe group (48.7% vs. 36.9%, *p* = 0.010). The length of hospital stay was significantly higher in severe patients as compared to non-severe patients (14.0 days (IQR 10.0–20.0) vs. 8.0 days (IQR 5.0–11.0), *p* < 0.001).

In terms of clinical presentation, our results revealed that significantly higher proportions of patients had a fever (93.5% vs. 74.5%, *p* < 0.001) and frequent onsets of dyspnea (30.5% vs. 13.9%, *p* < 0.001) in the severe group compared to the non-severe group. Mild symptoms such as fatigue (12.3% vs. 5.8%, *p* = 0.026), sore throat (10.2% vs. 3.9%, *p* = 0.016), headache (11.4% vs. 4.5%, *p* = 0.013) and abdominal pain (8.8% vs. 2.6%, *p* = 0.010) were significantly more common in the non-severe group than in the severe group. The detailed patient characteristics are presented in Table 1

### 3.2. Vitals and Laboratory Characteristics

**Figure 1:**
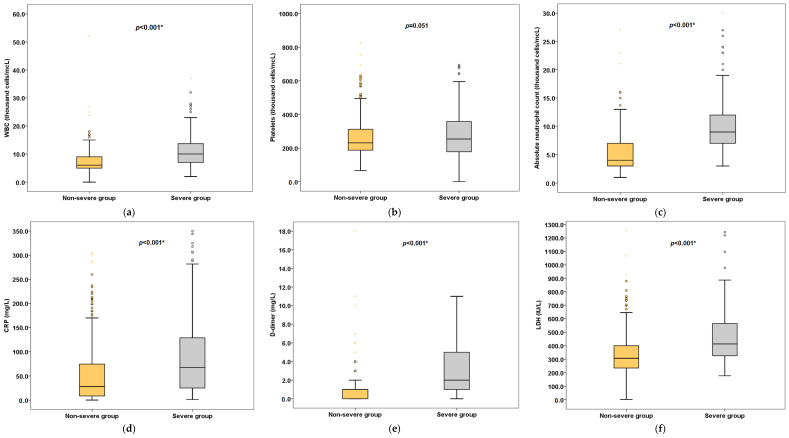
Laboratory results (selected) stratified by severity of disease. Box plots represent (**a**) WBC, (**b**) Platelets, (**c**) Neutrophils, (**d**) CRP, (**e**) D-dimer, (**f**) LDH. * Statistically significant.

Our results showed that temperature, heart rate and blood pressure did not significantly differ between severe and non-severe patients. However, the difference in respiratory rates between the two groups was statistically significant, with the respiratory rate being higher in the severe patients (24 breaths/min (IQR, 20–30) vs. 18 breaths/min (IQR, 18–22), *p* < 0.001) than in the non-severe patients. The vital signs and laboratory characteristics of COVID-19 patients stratified by severity of the disease are depicted in Table 2 and Figure 1.

As for the hematology investigations, hemoglobin (Hb) (11.9 g/dL (IQR, 10–13.1) vs. 14.0 g/dL (IQR, 12–15), *p* < 0.001) and red blood cell count (RBC) (4 × 10^6^/mcL (IQR, 4–5) vs. 5 × 10^6^/mcL (IQR, 4–5), *p* < 0.001) were significantly lower in severe patients than in non-severe patients. Severe patients had significantly higher neutrophil counts (9 × 10^3^/mcL (IQR, 7–12) vs. 4 × 10^3^/mcL (IQR, 3–7), *p* < 0.001) compared to non-severe patients. Additionally, in terms of biochemistry tests, severe patients had significantly higher levels of urea (9 mmol/L (IQR, 5–14) vs. 4.1 mmol/L (IQR, 3–7), *p* < 0.001), aspartate aminotransferase (AST) (50 IU/L (IQR, 30–86) vs. 39 IU/L (IQR, 26–54), *p* = 0.001), alanine aminotransferase (ALT) (54.5 IU/L (IQR, 35–96.5) vs. 44 IU/L (IQR, 44–75.2), *p* < 0.001) and blood glucose (8 mmol/L (IQR, 6–11) vs. 7 mmol/L (IQR, 6–10), *p* = 0.002).

Regarding the inflammatory markers, the levels of C-reactive protein (CRP) (67.5 mg/L (IQR, 25.0–130.8) vs. 28.0 mg/L (IQR, 8.5–74.7), *p* < 0.001), ferritin (673.5 ng/mL (IQR, 389.8–1489.5) vs. 439.5 ng/mL (IQR, 179.0–869.0), *p* < 0.001) and procalcitonin (2.0 ug/L (IQR, 1.0–5.0) vs. 0.0 ug/L (IQR, 0.0–0.02), *p* < 0.001) were significantly elevated in severe patients compared to in non-severe patients. Additionally, higher levels of coagulation indicators, prothrombin time (PT) (13 secs (IQR, 12–14) vs. 12 secs (11.0–13.0), *p* < 0.001) and D-dimer (2.0 mg/L (IQR, 1.0–5.0) vs. 1.0 mg/L (IQR, 0.0–1.0), *p* < 0.001) were reported in severe patients. Furthermore, the cardiac biomarkers, such as troponin, creatine kinase (CK) and creatine kinase-MB (CK-MB), were significantly higher in the severe group but within the normal limits. However, lactate dehydrogenase (LDH) (414 IU/L (IQR, 324–565) vs. 308 IU/L (IQR, 235.2–401), *p* < 0.001) and pro brain natriuretic peptide (proBNP) (204.5 ng/L (IQR, 60–892.8) vs. 71.0 ng/L (IQR, 27–195), *p* < 0.001) were significantly increased in patients with severe disease.

### 3.3. Risk Factors for Severity of Disease

To identify the risk factors associated with the severity of COVID-19, a univariable logistic regression analysis, including 50 key characteristics as independent variables, was performed. Laboratory variables were converted into categorical variables as per their reference ranges before including them in the model. Non-severe disease was the reference category in the univariable model. Thirty-six variables were found to be associated with severity of COVID-19 in the univariable model, as depicted in Table 3.

Age greater than 45 years (OR = 1.549, 95% CI: 1.052–2.283, *p* = 0.027); higher BMI (OR = 1.032, 95% CI: 1.004–1.061, *p* = 0.023); male gender (OR = 1.994, 95% CI: 1.309–3.037; *p* = 0.001); comorbidities greater than two (OR = 1.668, 95% CI: 1.081–2.575, *p* = 0.021); presentation symptoms such as fever (OR = 4.935, 95% CI: 2.509–9.707, *p* < 0.001), shortness of breath (OR = 9.814, 95% CI: 5.852–16.457, *p* < 0.001) and dyspnea (OR = 2.716, 95% CI: 1.752–4.210, *p* < 0.001); RBC less than 4.5 × 10^6^/mcL (OR = 4.807, 95% CI: 3.244–7.122, *p* < 0.001); Hb less than 13 g/dL (OR = 4.124, 95% CI: 2.789–6.097, *p* < 0.001); white blood count (WBC) greater than 11 × 10^3^/mcL (OR = 4.303, 95% CI: 2.042–9.068, *p* < 0.001); neutrophils greater than 7 × 10^3^/mcL (OR = 10.085, 95% CI: 4.205–24.188, *p* < 0.001); lymphocytes less than 1 × 10^3^/mcL (OR = 1.351, 95% CI: 1.114–13.382, *p* = 0.033); platelets less than 150 × 10^3^/mcL (OR = 4.883, 95% CI: 2.646–9.011, *p* < 0.001); sodium less than 135 mmol/L (OR = 23.011, 95% CI: 6.715–78.847, *p* < 0.001); potassium less than 3.6 mmol/L (OR = 7.717, 95% CI: 3.782–15.748, *p* < 0.001); urea greater than 6.5 mmol/L (OR = 5.222, 95% CI: 3.517–7.754, *p* < 0.001); serum creatinine greater than 115 umol/L (OR = 2.452, 95% CI: 1.592–3.777, *p* < 0.001); AST greater than 37 IU/L (OR = 1.592, 95% CI: 1.082–2.342, *p* = 0.018); ALT greater than 63 (OR = 1.602, 95% CI: 1.096–2.342, *p* = 0.015); PT greater than 12.3 s (OR = 3.602, 95% CI: 2.442–5.312, *p* < 0.001); CK greater than 308 IU/L (OR = 2.144, 95% CI: 1.318–3.488, *p* = 0.002); CK-MB greater than 3.6 IU/L (OR = 2.945, 95% CI: 2.001–4.334, *p* < 0.001); troponin greater than 60 ng/L (OR = 2.313, 95% CI: 1.549–3.454, *p* < 0.001); proBNP greater than 126 ng/L (OR = 2.250, 95% CI: 1.528–3.314, *p* < 0.001); procalcitonin greater than 0.10 ug/L (OR = 3.981, 95% CI: 2.663–5.951, *p* < 0.001); D-dimer greater than 0.55 mg/L (OR = 1.763, 95% CI: 3.476–9.768, *p* < 0.001); ferritin greater than 388 ng/mL (OR = 2.636, 95% CI: 1.733–4.007, *p* < 0.001); CRP greater than 3 mg/L (OR = 5.067, 95% CI: 1.999–12.849, *p* < 0.001) and LDH greater than 227 IU/L (OR = 10.249, 95% CI: 3.699–28.401, *p* < 0.001) were associated with increased risks of COVID-19 severity in univariate logistic regression model. However, presentation symptoms such as fatigue, sore throat and headache were associated with decreased risks of COVID-19 severity.

Our results of multivariable logistic regression analysis showed that the risks of having severe disease were 2.070 (95% CI: 1.035–4.141, *p* = 0.040) times higher among patients belonging to the age group of greater than 45 years, when compared with patients of less than 45 years. Male patients had a 3.151 (95% CI: 1.524–6.515, *p* = 0.002)-times greater risk of having severe disease compared to female patients.

Patients with presentation symptoms such as fever (OR = 3.681, 95% CI: 1.340–10.112, *p* = 0.011) and shortness of breath/dyspnea (OR = 5.360, 95% CI: 2.691–10.677, *p* < 0.001) had a significantly higher risk of contracting severe COVID-19 than did patients with no such symptoms. However, patients with mild presentation symptoms such as fatigue (OR = 0.256, 95% CI: 0.077–0.853, *p* = 0.027), nausea or vomiting (OR = 0.313, 95% CI: 0.099–0.992, *p* = 0.048) were less likely to develop severe disease.

Laboratory parameters including an Hb less than 13 g/dL (OR=3.170, 95% CI: 1.511–6.650, *p* = 0.002), neutrophils greater than 7 × 10^3^/mcL (OR = 4.894, 95% CI: 1.666–14.373, *p* = 0.004), lymphocytes less than 1 × 10^3^/mcL (OR = 7.783, 95% CI: 1.006–60.198, *p* = 0.049), sodium less than 135 mmol/L (OR = 5.417, 95% CI: 1.050–27.953, *p* = 0.044), potassium less than 3.6 mmol/L (OR = 3.364, 95% CI: 1.028–11.012, *p*=0.045), urea greater than 6.5 mmol/L (OR = 3.368, 95% CI: 1.687–6.726, *p* = 0.001) and LDH greater than 227 IU/L (OR = 6.257, 95% CI: 1.609–24.325, *p* = 0.008) were found to be independent risk factors associated with severe COVID-19 (Table 4).

### 3.4. Pharmacotherapy for COVID-19 Patients

Different drugs were used for COVID-19 management depending on the clinical condition of the patients and the guideline recommendations during the study period. Antivirals, interferons, corticosteroids, antimalarials, antibiotics, antiparasitics, interleukin-6 inhibitors and cell-based therapy were used for COVID-19 management in the study. Among 585 of the study participants, the majority of the patients were on antiviral drugs (524, 89.6%) including remdesivir, favipiravir, lopinavir/ritonavir, oseltamivir and camostat mesylate followed by antibiotics (377, 64.4%) including doxycycline and azithromycin; corticosteroids (358, 61.2%) including dexamethasone, methylprednisolone, hydrocortisone and prednisone; antimalarial drugs (246, 42.1%) such as hydroxychloroquine, chloroquine; interferons (183, 31.3%) such as interferon alfa-2b, interferon-beta-b1 and peginterferon alfa-2a; antiparasitic drugs (61, 10.4%) such as ivermectin; cell-based therapy (21, 3.6%); and interlukin-6 inhibitors (8, 1.4%) such as tocilizumab.

Our results revealed that a significantly higher proportion of patients received antiviral drugs (96.8 % vs. 87.0 %, *p* = 0.001) in the severe group compared to the non-severe group. In addition to this, a significantly higher number of severe patients received corticosteroids (91.6% vs. 50.3%, *p* < 0.001) compared to non-severe patients. Regarding individual drugs, remdesivir and favipiravir were more likely to be prescribed to severe patients than to non-severe patients (15.6% vs. 6.0%, *p* < 0.001). Moreover, a significantly higher proportion of patients received dexamethasone (52.6% vs. 28.3%, *p* < 0.001) and methylprednisolone (56.5% vs. 21.8%, *p* < 0.001) in the severe group than in the non-severe group. Table 5 details the drug utilization pattern in the study population stratified by the severity of the disease.

## 4. Discussion

Currently, the number of COVID-19 cases and associated deaths have dropped significantly, globally [16] as well as in the UAE [17]. In the UAE, this is drop is attributed to high COVID-19 vaccination rates. The UAE has one of the world’s highest vaccination rates, with around 96% of the population having received one dose of vaccine and around 86% of the population fully vaccinated [19]. In addition to this, compliance with precautionary measures and strengthened medical facilities have resulted in improved circumstances, enhanced recovery rate and reduced mortality rate. However, the pandemic is far from over, and it is very important to make well-timed diagnoses and initiate effective treatment for COVID-19 patients based on the severity of the disease. Therefore, the present study investigated the clinical characteristics, risk factors for severity and pharmacotherapy of COVID-19 in the UAE population.

Out of the total 585 COVID-19 patients, the proportion of male patients was higher than female patients. This study finding is in accordance with other studies on COVID-19, which reported a similar gender distribution [20,21,22]. In terms of the severity of the disease, a significantly higher proportion of males was in the severe group than of females. Additionally, multivariable logistic regression analyses revealed that male patients had a significantly greater risk of having severe disease compared to female patients. This finding is supported by a global COVID-19 meta-analysis that identified the male gender as a risk factor for the development of severe disease [23]. This finding can be attributed to gender differences in the innate and adaptive immune systems, angiotensin-converting enzyme 2 (ACE2) receptors expression, comorbidities and socio-cultural and behavioral aspects, which may account for the female advantage in COVID-19 [23].

Furthermore, we report that that the median age of severe patients was significantly higher compared to of non-severe patients, and the multivariable logistic regression model also showed that older age was an independent risk factor for disease severity. These findings are in line with previous studies, which reported older age as a critical independent predictor of severity and mortality in COVID-19 [24,25,26]. This could be because of many reasons, such as an increased number of comorbidities with age, an ageing immune system and the prevalence of polypharmacy in older adults.

Our results revealed that nearly half of the study population had had at least one comorbidity, with diabetes mellitus being the most common followed by hypertension and cardiovascular diseases. A number of previous studies [20,25,27] have reported similar comorbidity distribution, wherein hypertension, diabetes and cardiovascular diseases were the most common comorbidities among COVID-19 patients. We also report that a significantly higher proportion of patients with comorbid diabetes were in the severe group. Moreover, univariable logistic regression analysis revealed that diabetic patients had a significantly higher risk of contracting severe COVID-19. Similar observations have been reported by a number of previous studies analyzing the association between severity of COVID-19 and diabetes [28,29]. The reason for this association is the syndromic nature of diabetes, wherein accompanying comorbidities such as obesity, hypertension and cardiovascular diseases, older age and hyperglycemia all contribute towards increasing the risk of severe COVID-19.

Fever was the most common symptom is our study population, followed by dry cough and shortness of breath. These clinical features of COVID-19 patients in our study were consistent with several previous reports [20,22,30] that reported fever as the predominant symptom. However, a recent study conducted in the UAE by Harbi et al. reported cough as the most common symptom in hospitalized COVID-19 patients. Furthermore, Wang et al. and Zayet et al. reported other symptoms such as diarrhea, nausea, smell and taste disorders [22,31]. These symptoms were less common in our study population. Symptoms such as fever and shortness of breath were present in a significantly higher proportion of patients in the severe group as compared to in the non-severe group. Similar clinical presentations were reported by a meta-analysis, where fever was associated with severe or critical disease [32] and by Sun et al., wherein severe subgroup of COVID-19 patients had more frequent dyspnea or shortness of breath compared to the non-severe subgroup [26]. Furthermore, our multivariable logistic regression model revealed that patients with presentation symptoms such as fever and shortness of breath/dyspnea had a significantly higher risk of severe COVID-19 as compared to patients with no such symptoms. Our results are in accordance with the studies that have identified fever as a risk factor of severe disease and poor patient outcomes [33,34], likewise with studies that reported dyspnea as an indicator for progression to severe disease [35,36]. However, we also report that patients with mild presentation symptoms such as fatigue, nausea or vomiting were less likely to develop severe disease. Our results showed that temperature, heart rate and blood pressure did not significantly differ between severe and non-severe patients. However, the difference in respiratory rates between the two groups was statistically significant, with respiratory rates being higher in the severe patients than in the non-severe patients. These results are in line with the findings of previous reports [37].

Regarding the hematological characteristics of the study population, we report that in severe patients, Hb and RBC were significantly lower, whereas WBC and neutrophil counts were significantly higher as compared to non-severe patients. A univariable model revealed that an Hb less than 13 g/dL, WBC greater than 11 × 10^3^/mcL, neutrophils greater than 7 × 10^3^/mcL, lymphocytes less than 1 × 10^3^/mcL and platelets less than 150 × 10^3^/mcL were associated with severe COVID-19. However, a multivariable model identified only an Hb less than 13 g/dL, neutrophils greater than 7 × 10^3^/mcL and lymphocytes less than 1 × 10^3^/mcL. Our results are consistent with the findings of previous studies that reported anemia [38], neutrophilia [39], lymphopenia [39] and thrombocytopenia [40] to be linked with severe COVID-19.

The association of anemia with severe disease can be attributed to the fact that, in the COVID-19 setting, there is respiratory compromise and an increase in oxygen demand; in such a situation, anemia can further reduce the oxygen supply to the tissues, leading to further complications and adverse outcomes. Neutrophilia is reported to predict adverse outcomes in COVID-19 patients, and the neutrophil–to–lymphocyte ratio has been recognized as a predictor for severe disease [41]. Dysregulated neutrophil activity might be the reason for the immune-mediated increased COVID-19 severity [42]. Lymphopenia has been reported to be a prevalent manifestation in patients with severe COVID-19 as the virus directly attacks the lymphocytes coupled with disordered inflammatory cytokine milieu, leading to increased lymphocyte apoptosis [43]. The association of thrombocytopenia with COVID-19 severity can be explained by irreversible platelet consumption during the hyper-inflammation and hyper-coagulable states of severe COVID-19. In addition to this, viral infection can decrease maturation and increase the apoptosis of platelets [44].

Many studies have reported that COVID-19 has a significant impact on liver related biomarkers [45,46]. Higher serum AST and ALT levels and lower serum albumin levels have been associated with COVID-19 severity [47]. Our univariable model also revealed that patients with AST levels greater than 37 IU/L and ALT levels greater than 63 IU/L had a significantly higher risk of severe disease as compared to patients with normal levels of these enzymes. Since elevated liver enzymes are associated with disease severity, liver functions should be monitored for COVID-19 patients.

Electrolyte abnormalities are common in COVID-19 patients. These electrolyte disturbances are known to have an impact on the prognosis of the disease. Our multivariable logistic regression analysis identified sodium levels less than 135 mmol/L and potassium levels less than 3.6 mmol/L as independent risk factors associated with severe COVID-19. These results are consistent with the findings of studies conducted by Tezcan et al. and Moreno-P et al. [48,49]. Tezcan et al. reported that hyponatremia was associated with unfavorable outcomes and was an independent factor related to mortality in COVID-19 patients [48]. Moreno-P et al. identified hypokalemia as a sensitive biomarker for severe COVID-19 and as an independent predictor of invasive mechanical ventilation [49]. Hachim et al. identified urea as one of the predictors of developing critical COVID-19 [50]. In our study also, a urea level greater than 6.5 mmol/L at presentation was independently associated with severe disease. A baseline assessment of electrolytes and urea can play a crucial role in evaluating the risk of severe COVID-19.

A number of studies reported associations of inflammatory markers with COVID-19 severity [51,52]. Similarly, in our study also, a CRP greater than 3 mg/L, ferritin greater than 388 ng/mL and procalcitonin greater than 0.10 ug/L were found to be associated with severe COVID-19 in the univariable model. Coagulation disorders are frequently reported in patients with severe COVID-19 [53]. Many studies have reported that D-dimer dynamics can gauge the severity of COVID-19 with elevated levels linked to poor outcomes in patients [54]. In our study, severe patients had significantly higher levels of coagulation indicators, PT and D-dimer. The elevated D-dimer level indicates hypercoagulability and may accentuate thrombosis in COVID-19. Furthermore, we report that the cardiac biomarker, an LDH greater than 227 IU/L, was found to be an independent risk factor of severe COVID-19. Our results are consistent with a pooled analysis of nine studies involving 1532 patients which reported that elevated LDH levels were linked with increased odds of developing severe disease in patients with COVID-19 [55].

In our study, the management of COVID-19 patients was conducted in accordance with the National Guidelines for Clinical Management and Treatment of COVID-19 [14] published by the National Clinical Committee for COVID-19 Management, Ministry of Health and Prevention, UAE. Our results revealed that different types of drugs were prescribed for the management of COVID-19 and its associated complications in the study, namely: antiviral drugs, interferons, corticosteroids, antimalarial drugs, antibiotics, antiparasitic drugs, interleukin-6 inhibitors and cell-based therapy. A recent study conducted in the UAE reported similar pharmacotherapy pattern in hospitalized COVID-19 patients [56]. The majority of the patients in our study were on antivirals and corticosteroids. These results are similar to the drug-utilizing study conducted by Sun et al. in which majority of the COVID-19 patients were prescribed antivirals and glucocorticoids [26].

The most frequently prescribed antiviral in our study was favipiravir, followed by lopinavir/ritonavir. Favipiravir is a novel RNA-dependent RNA polymerase inhibitor with reported antiviral properties against a broad array of RNA viruses. Cai et al. and Chen et al. studied the effects of favipiravir against other antivirals in COVID-19 [57,58]. Cai et al. reported that favipiravir treated COVID-19 patients had better radiological improvement and quicker viral clearance as compared to lopinavir/ritonavir treated patients [57]. Furthermore, Chen et al. concluded that favipiravir was associated with a significantly shortened time to fever and cough reduction compared to umifenovir in COVID-19 [58]. Remdesivir, the only US-FDA-approved antiviral drug for the treatment of hospitalized COVID-19 patients on supplemental oxygen, was found to be effective in shortening the recovery time in hospitalized COVID-19 patients [59]. In our study, 50 patients fulfilling the treatment criteria as per the national guidelines were prescribed with remdesivir.

Severe COVID-19 patients can develop lung injury and multi-organ dysfunction because of systemic inflammatory response. Corticosteroids owing to their potent anti-inflammatory effects can play a key role in preventing and mitigating these effects. The RECOVERY trial reported that dexamethasone improved survival in ventilated and oxygen-receiving COVID-19 patients [60]. In our study also, a significantly higher proportion of severe patients received low dose dexamethasone compared to non-severe patients. Additionally, overall, out of the total 154 severe patients, 141 received corticosteroids for the management of COVID-19. These results are consistent with the findings of Lin et al. [61].

Interferons, a family of cytokines, with in vitro and in vivo antiviral properties, are proposed as a potential treatment for COVID-19. Studies have reported the clinical benefit of interferons IFN-β1b, IFN-α-2b and PEG IFN-α2b in severe COVID-19 [62,63,64]. In our study, nearly one-third of the patients received interferon therapy with IFN-β1b, IFN-α-2b and PEG IFN-α2b. A significantly higher proportion of severe patients received interferon therapy as compared to non-severe patients. This finding is attributed to the fact that interferon therapy has been shown to improve clinical outcomes in severely ill COVID-19 patients.

Tocilizumab, an interleukin-6 inhibitor, has been reported to minimize the risk of death in hospitalized severe-COVID-19 patients [65]. It has been approved in combination with dexamethasone by the US-FDA for use in certain severe hospitalized patients showing rapid respiratory decompensation. Likewise, in our study also, nine patients in the severe group received this drug for the management for their severe condition.

Several studies have suggested increased risks of thromboembolic manifestations associated with COVID-19. Guidelines recommend that hospitalized COVID-19 patients should receive prophylactic-dose anticoagulation [13,14]. In line with these guidelines, most of the patients in our study were given prophylactic-dose anticoagulant. Enoxaparin, the preferred low-molecular weight heparin for anticoagulation, was the most frequently prescribed medication in our study.

Early in the pandemic, the COVID-19 disease process was not clearly understood, and symptomatic and supportive care were the only available management options. During that time, there was an increased use of hydroxychloroquine and chloroquine in COVID-19 patients despite no rigorous evidence for efficacy. Since then, chloroquine and hydroxychloroquine, with or without azithromycin, have been examined for the treatment of COVID-19 in a number of clinical trials. Many RCTs and observational studies reported a lack of benefit and potential for toxicity with hydroxychloroquine or chloroquine in the management of COVID-19 [66,67,68]. Likewise, initially in our study, hydroxychloroquine and chloroquine with or without azithromycin were prescribed to the non-severe group of patients.

We found that the pharmacotherapy of COVID-19 patients in our study was diverse, and most of the medications were prescribed considering the clinical condition of the patients, including patients’ characteristics, disease severity, age and comorbidities. This drug utilization pattern of our study is consistent with previous studies that evaluated treatment patterns in COVID-19 patients [26,61]. The varied prescription pattern reported by different studies including ours is attributed to the following factors; firstly, the spectrum of pharmacotherapies for the management of COVID-19 is rapidly growing and evolving, and secondly, with time there are evolution and revisions to the treatment guidelines depending on the best available data at the given time point.

Our study had some limitations. First, the causal inference of the identified relationships is limited due to the cross-sectional study design. Second, data for some of the laboratory parameters of patients were missing. Although the missing data was replaced by an imputation technique, their role might not be estimated aptly in determining the severity of the disease. Thirdly, being a single-center study, the findings cannot be generalized and need to be validated in a larger patient population.

## 5. Conclusions

In our study, older age, male gender, presentation symptoms such as fever and dyspnea, low Hb, neutrophilia, lymphopenia, hyponatremia, hypokalemia, elevated levels of urea and LDH were found to be independent risk factors for developing severe disease among COVID-19 patients. The pharmacotherapy of COVID-19 patients in our study was diverse, and the medications were prescribed based on the clinical condition of the patients in accordance with the national guidelines. Data from this study will contribute towards the early detection of patients at a high risk of developing critical illness and the optimization of treatments in this rapidly evolving pandemic.

## Figures and Tables

**Table 1:** Demographic and clinical characteristics of COVID-19 patients stratified by severity of disease.

Variables	All Patients (n = 585)	Severity of COVID-19		*p* Value
		Non-Severe Group (n = 431)	Severe Group (n = 154)	
Age, years, median (IQR)	49.0 (39.0–59.0)	48.0 (38.0–58.0)	52.0 (42.6–65.0)	**0.006**
Age group, n (%)				**0.026**
≤45 years	234 (40.0)	184 (42.7)	50 (32.5)	
>45 years	351 (60.0)	247 (57.3)	104 (67.5)	
Gender, n (%)				**0.001**
Female	199 (34)	163 (37.8)	36 (23.4)	
Male	386 (66)	268 (62.2)	118 (76.6)	
Ethnicity, n (%)				0.97
Arab	191(32.6)	149 (34.6)	42 (27.3)	
Non-Arabs	394 (67.4)	282 (65.4)	112 (72.7)	
Tobacco use, n (%)	38 (6.5)	29 (6.7)	9 (5.8)	0.893
Alcohol use, n (%)	26 (4.4)	20 (4.6)	6 (3.9)	0.842
BMI, kg/m^2^, median (IQR)	28 (25–33)	28 (25–32)	29 (25–33.6)	0.164
Comorbidities, n (%)				
Diabetes	234 (40)	159 (36.9)	75 (48.7)	**0.010**
Hypertension	214 (36.6)	150 (34.8)	64 (41.6)	0.135
Obesity	21 (3.6)	13 (3)	8 (5.2)	0.212
Cardiovascular disease	119 (20.3)	80 (18.6)	39 (25.3)	0.074
Renal disease	50 (8.5)	31 (7.2)	19 (12.3)	0.050
Respiratory disease	33 (5.6)	22 (5.1)	11 (7.1)	0.347
Autoimmune disease	5 (0.9)	4 (0.9)	1 (0.6)	0.747
Psychological disease	15 (2.6)	8 (1.9)	7 (4.5)	0.070
Immunosuppression	4 (0.7)	3 (0.7)	1 (0.6)	0.952
Others	73 (12.5)	58 (13.5)	15 (9.7)	0.231
No. of comorbidities, median (IQR)	1.0 (0.0–2.0)	1.0 (0.0–2.0)	1.0 (0.0–3.0)	0.061
No. of comorbidities, n (%)				**0.035**
None	200 (34.2)	157 (36.4)	43 (27.9)	
One to two	267 (45.6)	197 (45.7)	70 (45.5)	
More than two	118 (20.2)	77 (17.9)	41 (26.6)	
Length of hospital stay, days, median (IQR)	9.0 (6.0–14.0)	8.0 (5.0–11.0)	14.0 (10.0–20.0)	**<0.001**
Length of hospital stay, n (%)				**<0.001**
≤7 days	229 (39.1)	206 (47.8)	23 (14.9)	
>7 days	356 (60.9)	225 (52.2)	131 (85.1)	
Signs and symptoms, n (%)				
Fever	465 (79.5)	321 (74.5)	144 (93.5)	**<0.001**
Pneumonia	477 (81.5)	324 (75.2)	153 (99.4)	**<0.001**
Cough	417 (71.3)	304 (70.5)	113 (73.4)	0.503
Shortness of breath	316 (54)	181 (42)	135 (87.7)	**<0.001**
Dyspnea	107 (18.3)	60 (13.9)	47 (30.5)	**<0.001**
Fatigue	62 (10.6)	53 (12.3)	9 (5.8)	**0.026**
Myalgia	126 (21.5)	100 (23.2)	26 (16.9)	0.102
Rhinorrhea	19 (3.2)	17 (3.9)	2 (1.3)	0.112
Sore throat	50 (8.5)	44 (10.2)	6 (3.9)	**0.016**
Olfactory and taste disorder	36 (6.2)	32 (7.4)	4 (2.6)	**0.032**
Chest pain	67 (11.5)	53 (12.3)	14 (9.1)	0.284
Abdominal pain	42 (7.2)	38 (8.8)	4 (2.6)	**0.010**
Diarrhea	65 (11.1)	57 (13.2)	8 (5.2)	**0.006**
Nausea or vomiting	80 (13.7)	74 (17.2)	6 (3.9)	**<0.001**
Headache	56 (9.6)	49 (11.4)	7 (4.5)	**0.013**
Chills	16 (2.7)	12 (2.8)	4 (2.6)	0.903
Wheezing	2 (0.3)	2 (0.5)	0	0.397
Rigors	1 (0.2)	1 (0.2)	0	0.550
Others	37 (6.3)	30 (7)	7 (4.5)	0.291

BMI: Body mass index. IQR: Inter quartile range. *p* values were calculated by χ^2^ test or Fisher’s exact test or two-sample median test, as appropriate. Statistically significant values are in bold.

**Table 2:** Vital signs and laboratory characteristics of study population stratified by severity of disease.

Variable	Severity of COVID-19		*p* Value
	Non-Severe Group (n = 431)	Severe Group (n = 154)	
Temperature (°C)			**0.004**
Sample size	431	154	
Median (IQR)	37 (37–38)	37 (37–38)	
Oxygen saturation (%)			0.158
Sample size	312	145	
Median (IQR)	97 (95–98)	96 (91.5–98)	
Respiratory rate (breaths/min)			**<0.001**
Sample size	431	154	
Median (IQR)	18 (18–22)	24 (20–30)	
Heart rate (beats/min)			0.863
Sample size	431	154	
Median (IQR)	88 (78–102)	88.5 (70–105)	
Systolic blood pressure (mmHg)			0.311
Sample size	431	154	
Median (IQR)	129 (116–144)	131 (114.75–145)	
Diastolic blood pressure (mmHg)			**<0.001**
Sample size	431	154	
Median (IQR)	78 (68–87)	73 (64–83.25)	
Laboratory parameters			
Red blood count (×10^6^/mcL)			**<0.001**
Sample size	431	154	
Median (IQR)	5 (4–5)	4 (4–5)	
Hemoglobin (gm/dL)			**<0.001**
Sample size	431	154	
Median (IQR)	14 (12–15)	11.9 (10–13.1)	
White blood count (×10^3^/mcL)			**<0.001**
Sample size	431	154	
Median (IQR)	6 (5–9)	10 (7–13.8)	
Absolute count			
Neutrophils (×10^3^/mcL)			**<0.001**
Sample size	428	153	
Median (IQR)	4 (3–7)	9 (7–12)	
Lymphocytes (×10^3^/mcL)			**<0.001**
Sample size	431	154	
Median (IQR)	1 (1–2)	1 (1–1)	
Platelet count (×10^3^/mcL)			0.051
Sample size	431	154	
Median (IQR)	231 (187–313)	254 (177–359)	
Sodium (mmol/L)			**0.005**
Sample size	431	154	
Median (IQR)	137 (134–139)	138 (136–141)	
Potassium (mmol/L)			0.131
Sample size	431	154	
Median (IQR)	4 (4–4)	4 (4–5)	
Calcium (mmol/L)			0.067
Sample size	427	152	
Median (IQR)	2 (2–2)	2 (2–2)	
Magnesium (mmol/L)			0.626
Sample size	430	153	
Median (IQR)	1 (1–1)	1 (1–1)	
Chloride (mmol/L)			**0.029**
Sample size	431	154	
Median (IQR)	101 (99–103)	102 (99–105)	
Urea (mmol/L)			**<0.001**
Sample size	431	154	
Median (IQR)	4.1 (3–7)	9 (5–14)	
Serum creatinine (umol/L)			0.28
Sample size	431	154	
Median (IQR)	81 (68–98)	89.5 (68–148.75)	
Uric acid (umol/L)			0.707
Sample size	430	154	
Median (IQR)	233 (168–312)	225.5 (145–303)	
Aspartate aminotransferase (IU/L)			**0.001**
Sample size	430	154	
Median (IQR)	39 (26–54)	50 (30–86)	
Alanine aminotransferase (IU/L)			**<0.001**
Sample size	430	154	
Median (IQR)	44 (44–75.2)	54.5 (35–96.5)	
Albumin (gm/L)			**<0.001**
Sample size	430	154	
Median (IQR)	32 (27–36)	23 (19.75–28)	
Total protein (gm/L)			**<0.001**
Sample size	429	154	
Median (IQR)	74 (70–79)	63 (56–72)	
Total bilirubin (umol/L)			**0.010**
Sample size	430	154	
Median (IQR)	8 (6–12)	9 (7–15)	
Prothrombin time (seconds)			**<0.001**
Sample size	419	150	
Median (IQR)	12 (11–13)	13 (12–14)	
Partial thromboplastin time (seconds)			**0.001**
Sample size	414	148	
Median (IQR)	34 (30–39)	37 (31–46.75)	
International normalized ratio (seconds)			**<0.001**
Sample size	422	149	
Median (IQR)	1 (1–1)	1 (1–1)	
Blood glucose (mmol/L)			**0.002**
Sample size	428	154	
Median (IQR)	7 (6–10)	8 (6–11)	
HbA1C (%)			0.26
Sample size	369	91	
Median (IQR)	6 (6–8)	7 (6–9)	
Creatine kinase (IU/L)			**0.001**
Sample size	378	118	
Median (IQR)	106.5 (61–197.5)	153 (76–407.75)	
Creatine kinase-MB (mcg/L)			**<0.001**
Sample size	329	81	
Median (IQR)	1 (0–1)	1 (0.7–2)	
Troponin (ng/L)			**0.002**
Sample size	394	137	
Median (IQR)	8 (5–17)	11.18 (6.06–27.45)	
Brain-type natriuretic peptide (ng/L)			**<0.001**
Sample size	353	128	
Median (IQR)	71 (27–195)	204.5 (60–892.75)	
Procalcitonin (ug/L)			**<0.001**
Sample size	417	151	
Median (IQR)	0 (0–0.02)	2 (1–5)	
D-dimer (mg/L)			**<0.001**
Sample size	425	149	
Median (IQR)	1 (0–1)	2 (1–5)	
Ferritin (ng/L)			**<0.001**
Sample size	428	146	
Median (IQR)	439.5 (179–869)	673.5 (389.75–1489.5)	
C-reactive protein (mg/L)			**<0.001**
Sample size	430	154	
Median (IQR)	28 (8.45–74.7)	67.5 (25–130.75)	
Lactate dehydrogenase (IU/L)			**<0.001**
Sample size	424	147	
Median (IQR)	308 (235.25–401)	414 (324–565)	

IQR: Inter quartile range. *p* values were calculated by Two-sample median test. Statistically significant values are in bold.

**Table 3:** Univariable logistic regression model of risk factors for severity of COVID-19.

Variable	Level	OR	B	95% CI	*p* Value
Age, years		1.026	0.025	1.013–1.039	**<0.001**
Age, years	≤45		Ref		
	>45	1.549	0.438	1.052–2.283	**0.027**
Body Mass Index, kg/m^2^	<25		Ref		
	≥25	1.052	0.051	0.676–1.637	0.821
Gender	Male	1.994	0.690	1.309–3.037	**0.001**
	Female		Ref		
No. of comorbidities		1.148	0.138	1.008–1.307	**0.038**
Number of comorbidities	≤2		Ref		
	>2	1.668	0.512	1.081–2.575	**0.021**
Type of comorbidities (No)					
Diabetes	Absent		Ref		
	Present	1.624	0.485	1.120–2.355	**0.011**
Hypertension	Absent		Ref		
	Present	1.332	0.287	0.914–1.942	0.136
Obesity	Absent		Ref		
	Present	1.762	0.566	0.716–4.336	0.218
Cardiovascular disease	Absent		Ref		
	Present	1.488	0.397	0.961–2.303	0.075
Renal disease	Absent		Ref		
	Present	1.816	0.597	0.993–3.320	0.053
Respiratory disease	Absent		Ref		
	Present	1.430	0.358	0.677–3.023	0.349
Autoimmune disease	Absent		Ref		
	Present	0.698	−0.360	0.077–6.291	0.748
Immunosuppression	Absent		Ref		
	Present	0.932	−0.070	0.096–9.032	0.932
Fever	Absent		Ref		
	Present	4.935	1.596	2.509–9.707	**<0.001**
Cough	Absent		Ref		
	Present	1.151	0.141	0.762–1.740	0.503
Shortness of breath/Dyspnea	Absent		Ref		
	Present	9.814	2.284	5.852–16.457	**<0.001**
Fatigue	Absent		Ref		
	Present	0.443	−0.815	0.213–0.921	**0.029**
Myalgia	Absent		Ref		
	Present	0.672	−0.397	0.417–1.084	0.103
Sore throat	Absent		Ref		
	Present	0.357	−1.031	0.149–0.854	**0.021**
Chest pain	Absent		Ref		
	Present	0.713	−0.338	0.384–1.326	0.285
Nausea or vomiting	Absent		Ref		
	Present	0.196	−1.632	0.083–0.459	**<0.001**
Headache	Absent		Ref		
	Present	0.371	−0.991	0.164–0.838	**0.017**
Red blood cells count (×10^6^/mcL)	≥4.5		Ref		
	<4.5	4.807	1.570	3.244–7.122	**<0.001**
Hemoglobin (gm/dL)	≥13		Ref		
	<13	4.124	1.417	2.789–6.097	**<0.001**
White blood cells count (×10^3^/mcL)	<4		Ref		
	4–11	1.282	0.249	0.621–2.645	0.501
	>11	4.303	1.459	2.042–9.068	**<0.001**
Neutrophils (×10^3^/mcL)	<2		Ref		
	2–7	2.209	0.792	0.906–5.385	0.081
	>7	10.085	2.311	4.205–24.188	**<0.001**
Lymphocytes (×10^3^/mcL)	<1	3.862	1.351	1.114–13.382	**0.033**
	1–3	1.302	0.264	0.372–4.566	0.680
	>3		Ref		
Platelet count (×10^3^/mcL)	<150	4.883	1.586	2.646–9.011	**<0.001**
	150–450	0.704	−0.352	0.440–1.126	0.142
	>450		Ref		
Blood glucose (mmol/L)	<3.9		Ref		
	3.9–6.1	0.718	−0.331	0.073–7.066	0.777
	>6.1	1.350	0.300	0.139–13.127	0.796
HbA1C (%)	<4.8		Ref		
	4.8–6	0.133	−2.015	0.010–1.804	0.130
	>6	0.808	−0.214	0.073–8.974	0.862
Sodium (mmol/L)	<135	23.011	3.136	6.715–78.847	**<0.001**
	135-145		Ref		
	>145	1.077	0.074	0.701–1.655	0.734
Potassium (mmol/L)	<3.6	7.717	2.043	3.782–15.748	**<0.001**
	3.6–5.1		Ref		
	>5.1	1.319	0.277	0.754–2.307	0.331
Calcium (mmol/L)	<2.6		Ref		
	≥2.6	0.938	−0.064	0.187–4.698	0.938
Urea (mmol/L)	≤6.5		Ref		
	>6.5	5.222	1.653	3.517–7.754	**<0.001**
Serum creatinine (umol/L)	≤115		Ref		
	>115	2.452	0.897	1.592–3.777	**<0.001**
Aspartate aminotransferase (IU/L)	≤37		Ref		
	>37	1.592	0.465	1.082–2.342	**0.018**
Alanine aminotransferase (IU/L)	≤63		Ref		
	>63	1.602	0.471	1.096–2.342	**0.015**
Total bilirubin (umol/L)	≤17		Ref		
	>17	1.492	0.400	0.894–2.489	0.125
Prothrombin time (secs)	≤12.3		Ref		
	>12.3	3.602	1.281	2.442–5.312	**<0.001**
Partial thromboplastin time (secs)	≤37.7		Ref		
	>37.7	2.161	0.770	1.476–3.163	**<0.001**
International normalized ratio (secs)	≤1.29		Ref		
	>1.29	5.969	1.787	2.709–13.153	**<0.001**
Creatine kinase (IU/L)	≤308		Ref		
	>308	2.144	0.763	1.318–3.488	**0.002**
Creatine kinase-MB (IU/L)	≤3.6		Ref		
	>3.6	2.945	1.080	2.001–4.334	**<0.001**
Troponin (ng/L)	≤60		Ref		
	>60	2.313	0.839	1.549–3.454	**<0.001**
Brain-type natriuretic peptide (ng/L)	≤126		Ref		
	>126	2.250	0.811	1.528–3.314	**<0.001**
Procalcitonin (ug/L)	≤0.10		Ref		
	>0.10	3.981	1.381	2.663–5.951	**<0.001**
D-dimer (mg/L)	≤0.55		Ref		
	>0.55	5.827	1.763	3.476–9.768	**<0.001**
Ferritin (ng/mL)	≤388		Ref		
	>388	2.636	0.969	1.733–4.007	**<0.001**
C-reactive protein (mg/L)	≤3		Ref		
	>3	5.067	1.623	1.999–12.849	**0.001**
Lactate dehydrogenase (IU/L)	≤227		Ref		
	>227	10.249	2.327	3.699–28.401	**<0.001**

OR: Odds ratio. CI: Confidence Interval. Statistically significant values are in bold.

**Table 4:** Multivariable logistic regression model of the risk factors for severity of COVID-19.

Variable	Level	OR	B	95% CI	*p* Value
Age, years	≤45		Ref		
	>45	2.070	0.728	1.035–4.141	**0.040**
Gender	Male	3.151	1.148	1.524–6.515	**0.002**
	Female		Ref		
Number of comorbidities	≤2		Ref		
	>2	1.816	0.597	0.873–3.777	0.110
Type of comorbidities (No)					
Diabetes	Absent		Ref		
	Present	0.624	0.152	0.634–2.139	0.624
Fever	Absent		Ref		
	Present	3.681	1.303	1.340–10.112	**0.011**
Shortness of breath/Dyspnea	Absent		Ref		
	Present	5.360	1.679	2.691–10.677	**<0.001**
Fatigue	Absent		Ref		
	Present	0.256	−1.364	0.077–0.853	**0.027**
Sore throat	Absent		Ref		
	Present	0.695	−0.364	0.215–2.246	0.543
Nausea or vomiting	Absent		Ref		
	Present	0.313	−1.161	0.099–0.992	**0.048**
Headache	Absent		Ref		
	Present	0.336	−1.090	0.095–1.193	0.092
Red blood cells count (×10^6^/mcL)	≥4.5		Ref		
	<4.5	1.690	0.524	0.828–3.450	0.150
Hemoglobin (gm/dL)	≥13		Ref		
	<13	3.170	1.154	1.511–6.650	**0.002**
White blood cells count (×10^3^/mcL)	<4		Ref		
	4–11	0.813	−0.207	0.248–2.664	0.733
	>11	1.438	0.363	0.409–5.053	0.571
Neutrophils (×10^3^/mcL)	<2		Ref		
	2–7	1.830	0.604	0.621–5.391	0.273
	>7	4.894	1.588	1.666–14.373	**0.004**
Lymphocytes (×10^3^/mcL)	<1	7.783	2.052	1.006–60.198	**0.049**
	1–3	4.411	1.484	0.563–34.554	0.158
	>3		Ref		
Platelet count (×10^3^/mcL)	<150	2.893	1.062	0.942–8.883	0.063
	150–450	0.659	−0.417	0.323–1.343	0.251
	>450		Ref		
Sodium (mmol/L)	<135	5.417	1.690	1.050–27.953	**0.044**
	135–145		Ref		
	>145	1.147	0.137	0.596–2.208	0.682
Potassium (mmol/L)	<3.6	3.364	1.213	1.028–11.012	**0.045**
	3.6–5.1		Ref		
	>5.1	1.159	0.148	0.478–2.811	0.744
Urea (mmol/L)	≤6.5		Ref		
	>6.5	3.368	1.214	1.687–6.726	**0.001**
Serum creatinine (umol/L)	≤115		Ref		
	>115	0.426	−0.854	0.167–1.088	0.074
Aspartate aminotransferase (IU/L)	≤37		Ref		
	>37	1.171	0.158	0.601–2.283	0.643
Alanine aminotransferase (IU/L)	≤63		Ref		
	>63	0.782	−0.245	0.404–1.517	0.467
Prothrombin time (secs)	≤12.3		Ref		
	>12.3	1.035	0.035	0.550–1.950	0.915
Partial thromboplastin time (secs)	≤37.7		Ref		
	>37.7	0.677	−0.389	0.347–1.322	0.253
International normalized ratio (secs)	≤1.29		Ref		
	>1.29	1.100	0.096	0.259–4.680	0.897
Creatine kinase (IU/L)	≤308		Ref		
	>308	1.039	0.038	0.466–2.316	0.925
Creatine kinase-MB (IU/L)	≤3.6		Ref		
	>3.6	1.708	0.536	0.915–3.189	0.093
Troponin (ng/L)	≤60		Ref		
	> 60	1.117	0.111	0.541–2.305	0.765
Brain-type natriuretic peptide (ng/L)	≤126		Ref		
	>126	0.952	−0.050	0.496–1.825	0.881
Procalcitonin (ug/L)	≤0.10		Ref		
	>0.10	1.379	0.321	0.671–2.833	0.382
D-dimer (mg/L)	≤0.55		Ref		
	>0.55	1.322	0.279	0.646–2.703	0.445
Ferritin (ng/mL)	≤388		Ref		
	>388	0.615	−0.486	0.302–1.251	0.180
C-reactive protein (mg/L)	≤3		Ref		
	>3	0.999	−0.001	0.271–3.690	0.999
Lactate dehydrogenase (IU/L)	≤227		Ref		
	>227	6.257	1.834	1.609–24.325	**0.008**

OR: Odds ratio. CI: Confidence Interval. Statistically significant values are in bold.

**Table 5:** Pharmacotherapy of COVID-19 patients stratified by severity of the disease.

Drugs	ATC	All Patients (n = 585)	Severity of COVID-19		*p* Value
			Non-Severe Group (n = 431)	Severe Group (n = 154)	
Antivirals		524 (89.6)	375 (87)	149 (96.8)	**0.001**
Favipiravir	J05AX27	409 (69.9)	278 (64.5)	131 (85.1)	**<0.001**
Lopinavir/Ritonavir	J05AR10	182 (31.1)	143 (33.2)	39 (25.3)	0.071
Remdesivir	J05AB16	50 (8.5)	26 (6)	24 (15.6)	**<0.001**
Oseltamivir	J05AH02	3 (0.5)	3 (0.7)	0 (0.0)	0.299
Camostat mesylate	B02AB04	72 (12.3)	42 (9.7)	30 (19.5)	**0.002**
Corticosteroids		358 (61.2)	217 (50.3)	141 (91.6)	**<0.001**
Dexamethasone	H02AB02	203 (34.7)	122 (28.3)	81 (52.6)	**<0.001**
Methylprednisolone	H02AB04	181 (30.9)	94 (21.8)	87 (56.5)	**<0.001**
Hydrocortisone	H02AB09	33 (5.6)	12 (2.8)	21 (13.6)	**<0.001**
Prednisone	H02AB07	31 (5.3)	24 (5.6)	7 (4.5)	0.627
Betamethasone	H02AB01	1 (0.2)	0 (0.0)	1 (0.6)	0.094
Interleukin-6 Inhibitors		8 (1.4%)	2 (0.5%)	6 (3.9%)	**<0.001**
Tocilizumab	L04AC07	11 (1.9)	2 (0.5)	9 (5.8)	**<0.001**
Interferons		183 (31.3)	102 (23.7)	81 (52.6)	**<0.001**
Interferon beta-1b	L03AB08	65 (11.1)	34 (7.9)	31 (20.1)	**<0.001**
Interferon alfa-2b	L03AB05	115 (19.7)	70 (16.2)	45 (29.2)	**0.001**
Peginterferon alfa-2a	L03AB11	12 (2.1)	4 (0.9)	8 (5.2)	**0.001**
Cell-based therapy		21 (3.6)	15 (3.5)	6 (3.9)	0.812
Stem cells	B05AX04	21 (3.6)	15 (3.5)	6 (3.9)	0.812
Antimalarial drugs		246 (42.1)	194 (45)	52 (33.8)	**0.015**
Hydroxychloroquine	P01BA02	239 (40.9)	190 (44.1)	49 (31.8)	**0.008**
Chloroquine	P01BA01	41 (7)	31 (7.2)	10 (6.5)	0.771
Antiparasitic drugs		61 (10.4)	31 (7.2)	30 (19.5)	**<0.001**
Ivermectin	P02CF01	61 (10.4)	31 (7.2)	30 (19.5)	**<0.001**
Antibiotics		377 (64.4)	256 (60.1)	118 (76.6)	**<0.001**
Azithromycin	J01FA10	78 (13.3)	60 (13.9)	18 (11.7)	0.484
Doxycycline	J01AA02	339 (57.9)	231 (53.6)	108 (70.1)	**<0.001**
Anticoagulants		562 (96.1)	409 (94.9)	153 (99.4)	**0.015**
Enoxaparin	B01AB05	558 (95.4)	406 (94.2)	152 (98.7)	**0.022**

ATC: Anatomical Therapeutic Chemical code. *p* values were calculated by χ^2^ test or Fisher’s exact test. Statistically significant values are in bold.

## Data Availability

Data will be available from the corresponding author upon reasonable request.
